# Phytotoxic Metabolites Isolated from *Neufusicoccum batangarum*, the Causal Agent of the Scabby Canker of Cactus Pear (*Opuntia ficus-indica* L.)

**DOI:** 10.3390/toxins12020126

**Published:** 2020-02-18

**Authors:** Marco Masi, Francesco Aloi, Paola Nocera, Santa Olga Cacciola, Giuseppe Surico, Antonio Evidente

**Affiliations:** 1 Dipartimento Scienze Chimiche, Università di Napoli Federico II, Complesso Universitario Monte S. Angelo, Via Cintia 4, 80126 Napoli, Italy; marco.masi@unina.it (M.M.); paola.nocera@unina.it (P.N.); 2 Dipartimento di Scienze Agrarie, Alimentari, Forestali e Ambientali, Università di Palermo, V. le delle Scienze 4, 90128 Palermo, Italy; francesco.aloi@unipa.it; 3 Dipartimento di Agricoltura, Alimentazione e Ambiente, Università di Catania, Via Santa Sofia 100, 95123 Catania, Italy; 4 Dipartimento di Scienze e Tecnologie Agrarie, Alimentari, Ambientali e Forestali, Sez. Patologia vegetale ed entomologia, Università di Firenze, Piazzale delle Cascine 28, 50144 Firenze, Italy; giuseppe.surico@unifi.it

**Keywords:** cactus pear, scabby cankers, *Neofusicoccum batangarum*, phytotoxins

## Abstract

Six phytotoxins were obtained from the culture filtrates of the ascomycete *Neofusicoccum batangarum*, the causal agent of the scabby canker of cactus pear (*Opuntia ficus-indica* L.) in minor Sicily islands. The phytotoxins were identified as (−)-(*R*)-mellein (**1**); (±)-botryoisocoumarin A (**2**); (−)-(3*R*,4*R*)- and (−)-(3*R*,4*S*)-4-hydroxymellein (**3** and **4**); (−)-terpestacin (**5**); and (+)-3,4-dihydro-4,5,8-trihydroxy-3-methylisocoumarin, which we named (+)-neoisocoumarin (**6**). This identification was done by comparing their spectral and optical data with those already reported in literature. The absolute configuration (3*R*,4*S*) to (+)-neoisocoumarin (**6**) was determined using the advanced Mosher method. All six metabolites were shown to have phytotoxicity on the host (cactus pear) and non-host (tomato) plants, and the most active compounds were (±)-botryoisocoumarin A (**2**), (−)-terpestacin (**5**), and (+)-neoisocoumarin (**6**).

## 1. Introduction

Cactus pear (*Opuntia ficus-indica* (L.) Mill.), Cactaceae family, is believed to be native of Mexico [1], and after the discovery of America, it was introduced into the Mediterranean Basin where it is now naturalized [2]. In Sicily, it has become an economically important fruit crop and a characteristic feature of the landscape. Cactus pear is also cultivated, mostly as productive living fences, in minor islands around Sicily, and as a fruit crop in Sardinia, Apulia, Calabria, and Basilicata Italian regions. Countries where cactus pear is also cultivated include Mexico, United States of America, Chile, Brazil, North Africa, South Africa, Middle East, Turkey, Tunisia, Malta, etc.

**Figure 1:**
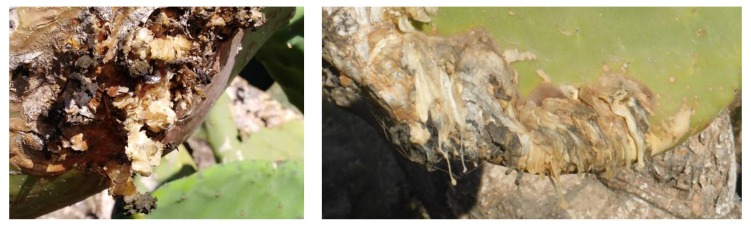
Symptoms of scabby cankers on cladodes of pear cactus (*O. ficus-indica* L.), including radially expanding, crusty, concentric, silvery, perennial cankers, with a leathery, brown halo (**left panel**); and an abundant milky viscous exudate, caking on contact with air, which leaked from cankers and formed strips or cerebriform masses (**right panel**).

Recently, a severe infectious disease of the plant was reported in some minor islands of Sicily: Lampedusa and Linosa of the Pelagie archipelago; Favignana of the Aegadian archipelago; and Ustica, a small island in the Tyrrhenian Sea [3]. Symptoms of this disease, which has been named scabby cankers, were visible on cladodes and included radially expanding, crusty, concentric, silvery, perennial cankers, with a leathery, brown halo (Figure 1). Characteristically, an abundant, milky, viscous exudate, caking on contact with air, leaked from cankers and formed strips or cerebriform masses (Figure 1). With time, the exudate in the central part of the canker became black, giving the cankers an appearance of carbonaceous crusts, and the cankers ceased to expand in the coldest season of the year.

The causal agent of the scabby canker of cactus pear found in minor islands of Sicily was identified as *Neofusicoccum batangarum* Begoude, Jol. Roux & Slippers, a fungal species not reported previously in Europe, whose distribution includes Africa, Brazil, and USA [4,5,6]. In Brazil, *N. batangarum* was reported as an aggressive pathogen of cashew (*Anacardium occidentale*) and cochineal cactus (*Nopalea cochenillifera* (L.) Salm-Dyck, syn. *Opuntia cochenillifera* (L.) Mill.), a relative of cactus pear [6,7]. On cochineal cactus in Brazil, *N. batangarum*, alone or in association with other fungi, including different species of Bothryosphaeriaceae, causes a disease that was named cladode brown spot and constitutes a serious threat to the cultivation of cochineal cactus as a fodder for livestock [7,8,9,10]. In preliminary cross-pathogenicity tests, the fungus isolated from infected cactus pear plants in minor islands of Sicily was able to reproduce the disease symptoms on the host plant and to induce a disease reaction on other plant species, such as Aleppo pine (*Pinus halepensis*), almond (*Prunus dulcis*), sweet orange (*Citrus sinensis*), citrange (*Citrus sinesis Poncirus trifoliata*), and holm oak (*Quercus ilex*) trees. These results show that *N. batangarum* has a wide host range like many other Botryosphaeriaceae species, which are frequently reported as causal agents of different important crop diseases, including the grapevine Botryosphaeria dieback [11,12,13,14,15].

The purpose of this study was to characterize the agent of the scabby canker of cactus pear for its ability to accumulate in culture biologically active substances, namely phytotoxins, which may have a role in the production of disease symptoms. The chemical identification of phytotoxins, obtained from the culture filtrates of *N. batangarum*, and their toxic effects on host and non-host plants are reported.

## 2. Results and Discussion

**Figure 2:**
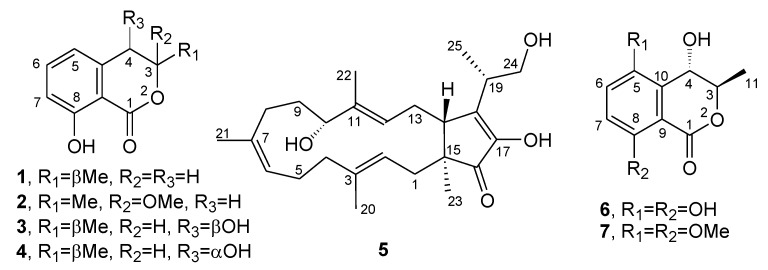
The structures of (−)-(*R*)-mellein (**1**), (±)-botryoisocoumarin A (**2**), (−)-(3*R*,4*R*)- and (−)-(3*R*,4*S*)-hydroxymellein (**3** and **4**), (−)-terpestacin (**5**), and (+)-neoisocoumarin (**6**).

The organic extract of the *N. batangarum* culture was purified using both CC (Column Chromatography) and TLC (Thin Layer Chromatography) as reported in detail in the Materials and Methods, to produce five pure metabolites. They were identified, by comparing their ^1^H NMR, MS, and specific optical rotation with the data reported in the literature, as (−)-(*R*)-mellein; (±)-botryosisocoumarin A; (−)-(3*R*,4*R*)- and (−)-(3*R*,4*S*)-4-hydroxymellein; (−)-terpestacin; and (+)-3,4-dihydro-4,5,8-trihydroxy-3-methylisocoumarin, which was named (+)-neoisocoumarin (**1**–**6**; Figure 2). The purity of compounds **1**–**6** was >95% as ascertained via ^1^H NMR and HPLC analysis.

These data were very similar to those previously reported in the literature for **1** [13,16,17,18,19,20], for **2** isolated in a racemic mixture [16], for **3** and **4 [17,21,22]** for **5 [23,24,25]**, and for **6 [26]**. Furthermore, **1** and **3**–**5** were also identified using TLC, performed in different conditions, in comparison using co-chromatography with standards.

(−)-(*R*)-Mellein (**1**) and its derivatives, including **3** and **4**, belong to the class of isocoumarins and are widely found in the fungal kingdom [13,15,19,20,21,27,28,29,30,31]; they have also been produced by plants and insects [32,33,34,35]. They are reported as phytotoxic metabolites of the fungi pathogen of forest and ornamental plants [36,37], and have been shown to possess a plethora of biological activities, including antiviral and antiparasitic activities [27,38,39,40]. Recently, in an advanced review of phytotoxins produced by grapevine pathogens [15], **1**, **3**, and **4** are reported as known phytotoxic metabolites synthesized by Botryosphaeriaceae species, inducing grapevine trunk disease, and **1** has also been found in infected grapevine wood [20]. These results suggested the potential use of these compounds as predictive biomarkers for early recognition of the disease [15]. However, the role of **1**, **3**, and **4** in the pathogenic process has not yet been clarified. Considering the potential application, some isocoumarins, such as chenisocoumarin, have been tested for the control of noxious weeds [41,42].

(±)-Botryoisocoumarin A (**2**) was isolated, together with 3,8-dihydroxy-3-methylisochroman-1-one and 4,8-dihydroxy-3-methylisochroman-1-one from *Botryosphaeria* sp. F00741 [16]. Then, it was also obtained together with five other metabolites from the mangrove *Kandelia candel*. This is an endophytic fungus *Botryopspheria* sp. KcF6 studied during a screening carried out to find new metabolites for drug development. Compound **2** showed COX-2 inhibitory activity (IC_50_ = 6.51 µM) but no cytotoxic activity. However, the structure drawn did not correspond to that of **2 [43]**. Successively, **2** was isolated from the marine mangrove-derived fungus *Aspergillus ochraceus*, together with three new metabolites and another eleven known ones. In this study, some efforts were made to assign its absolute configuration (AC) at C-3 using X-ray methods but the results demonstrated the racemic nature of **2**, which was then named (±)-botryoisocoumarin A [44]. Compound **2**, together with a new acetate derivative and four already known compounds, were also isolated from *Aspergillus westerdijkiae* SCSIO 05233, a deep see fungus, but **2** did not show antibiotic or cytotoxic activities [45].

(−)-Terpestacin (**5**) was isolated from the endophytic fungus *F. proliferatum* MA-84 [46] and from *Cleistothelebolus nipigonensis* and *Neogymnomyces virgineus* [25]. In addition, some key derivatives were hemi-synthetized from terpestacin and fusaproliferin. When they were tested against *Alternaria brassicicola*, *Botrytis cinerea* and *Fusarium graminearum* showed antifungal activity [25]. Recently, terpestacin was also isolated from *Rutstroemia capillus-albis* (Rutstroemiaceae, Helotiales, Leotiomycetes), the causal agent of “bleach blonde syndrome” on the grass weed *Bromus tectorum* (cheatgrass) in North America [47]. When assayed on the host plant, **5** showed high phytotoxicity at 10^-4^ M, thus they should have a role in pathogenesis on *B. tectorum* [47].

(+)-3,4-Dihydro-4,5,8-trihydroxy-3-methylisocoumarin (**6**) was previously obtained, together with two already known compounds, from *Phomopsis* sp. (No. ZH-111) during a screening aimed at finding new compounds from endophytes of the South China Sea [26]. When assayed on zebrafish, the tetrasubstituted-3,4-dihydroisocoumarin significantly accelerates the growth of vessels but showed only weak cytotoxicity on the two cancer cell lines tested [26]. However, Yang et al. [26] assigned only the 3R*,4S* relative configuration to this compound based on the correlation measured in the NOESY spectrum. After recording the NOESY spectrum of **6** in the same conditions, we observed the expected correlations between H-6 and H-7, H-3 and Me-11, and H-4 and Me-11, and thus **6** had the same relative configuration that was assigned by Jang et al. [26].

Thus, to assign the absolute configuration at C-3 and C-4, **6** was firstly converted in the corresponding 5,8-*O,O′*-dimethyl ether derivative (**7**), whose spectroscopic data (^1^H and ^13^C NMR and ESI MS) were fully consistent with the structure of **6**. The ^1^H NMR spectrum of **7** differed from that of **6** due to the two methoxy groups at δ 3.90 and 3.88. Its ESI MS spectrum exhibited the dimer sodiated form [2M + Na]^+^, the potassium [M + K]^+^ and sodium [M + Na]^+^ clusters, and the protonated form [M + H]^+^ at *m/z* 499, 277, 261, and 239, respectively. Furthermore, the protonated form generated the significant fragmentation ion [M + H - H_2_O]^+^ at *m*/*z* 193 via the loss of H_2_O.

**Figure 3:**
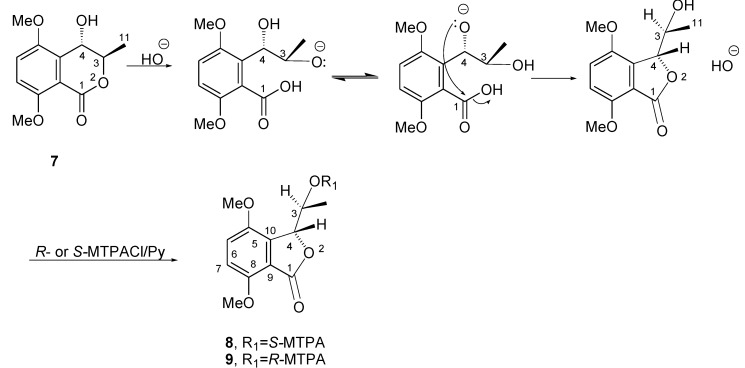
Mechanism of the conversion of the dimethylether of (+)-neoisocoumarin (**7**) into the corresponding diastereomeric benzofuranones (**8** and **9**) by reaction with *R*-(−)-α-methoxy-α-trifluoromethylphenylacetyl (MTPA) and *S*-(+)-MTPA chlorides.

5,8-*O,O′*-dimethyl ether derivative of **6** (**7**) was transformed into the relative diastereomeric *S*-MTPA and *R*-MTPA monoesters (**8** and **9**, respectively) by reacting with *R*-(−)-α-methoxy-α-trifluoromethylphenylacetyl (MTPA) and *S*-(+)MTPA chlorides. Surprisingly, the downfield shifts of H-3 (Δδ 0.48 and 0.47 in both **8** and **9**, respectively), instead of the expected downfield shift of H-4,was observed by comparing the ^1^H NMR spectra of **8** and **9** with that of **7**. The reaction mechanism that could explain this result is reported in Figure 3. The driving force of the reaction was the hydroxyl anion generated from KOH where pyridine was preserved in non-dry conditions.

**Figure 4:**
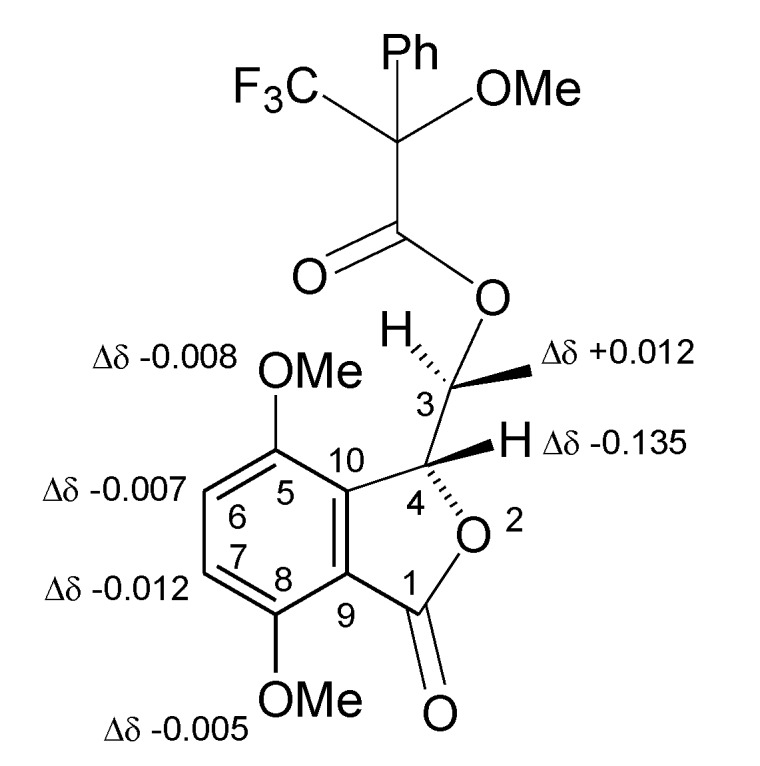
Structures of 4-*O*-*S*- and 4-*O*-*R*-MTPA esters of 5,8-*O*,*O*′-dimethyl ether of (+)-neoisocoumarin (**8** and **9**, respectively), reporting the Δδ value of each proton system.

However, the stereochemistry of C-3 and C-4 in the resulting benzofuranone did not change. The intermediate benzofuranone reacted with (*R*)-(−)- and (*S*)-(+) MTPACl, yielding the corresponding monoesters **8** and **9**, respectively. Subtracting the chemical shifts of **9** from those of **8** (Table 1), the Δδ (**8**–**9**) values for all the protons were determined and reported in Figure 4. 

Appling model A as reported in Cimmino et al. [48], the (*R*) configuration was assigned at C-3, and consequently, on the basis of NOESY data, the (*S*) one was at C-4. Then, **6** was formulated as (+)-(3*R*,4*S*)-3,4 dihydro-4,5,8-trihydroxy-3-methylisocoumarin and named (+)-neoisocumarin.

**Figure 5:**
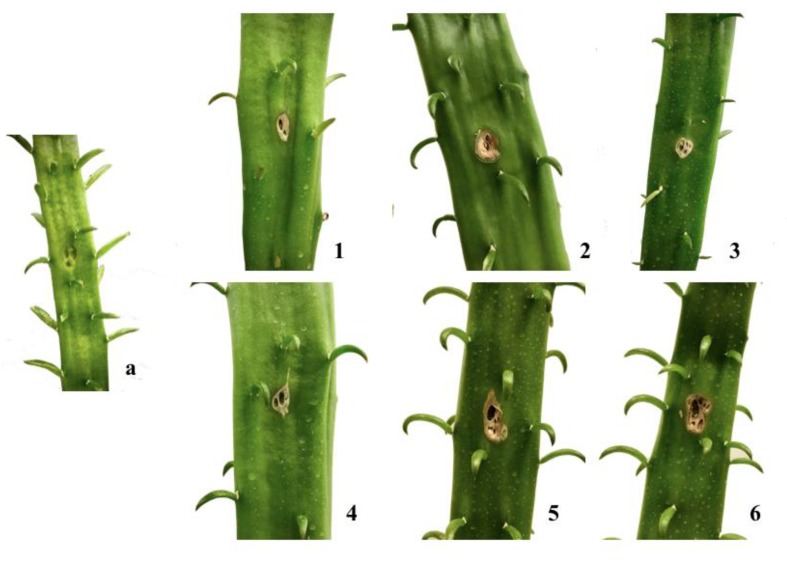
Control cladode (MeOH 4%, *v*/*v*) (**a**), necrotic areas produced by (−)-(*R*)-mellein (**1**), (±)-botryoisocoumarin A (**2**), (−)-(3*R*,4*R*)-4-hydroxymellein (**3**), (−)-(3*R*,4*S*)-4-hydroxymellein (**4**), (−)-terpestacin (**5**), and (+)-neoisocoumarin (**6**) on cladodes of cactus pear.

**Figure 6:**
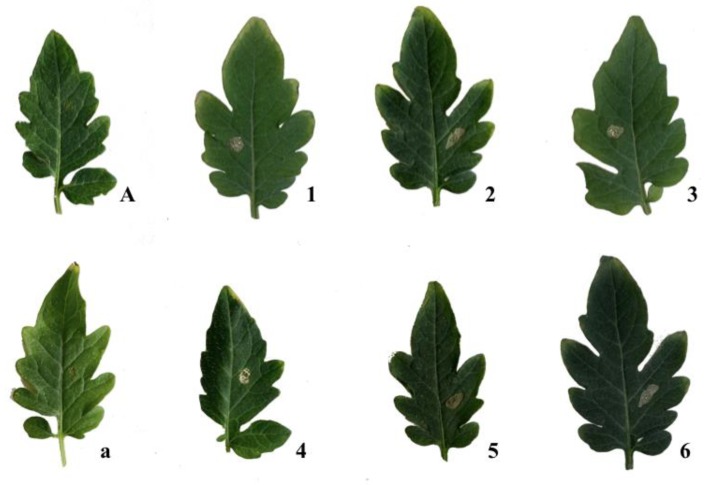
Control leaves with sterile distilled water (**A**) and MeOH (4% *v*/*v*) (**a**); necrotic areas produced by (−)-(*R*)-mellein (**1**), (±)-botryoisocoumarin A (**2**), (−)-(3*R*,4*R*)-4-hydroxymellein (**3**), (−)-(3*R*,4*S*)-4-hydroxymellein (**4**), (−)-terpestacin (**5**), and (+)-neoisocoumarin (**6**) on tomato leaves.

All the isolated compounds (**1**–**6**) were screened for phytotoxic activity as described in detail in Materials and Methods. All phytotoxins tested at the highest concentrations induced necrosis around inoculation points after 7 days, on cladodes of cactus pear (Figure 5), as well as on tomato leaves (Figure 6). However, **1**, **3**, and **4** were only phytotoxic at the highest concentrations on cactus pear and on non-host tomato plants (Table 2 and Table 3). All three metabolites were found to be produced by *N. parvum*, another species in the genus, and other fungi in the Botryospaeriaceae family associated with grapevine trunk diseases, confirming that they may be virulence factors in plant diseases caused by these fungi, although their role in the pathogenesis is still controversial [19,20]. (±)-Botryoisocoumarin A (**2**), (+)-neoisocoumarin (**6**), and (−)-terpestacin (**5**) showed phytotoxic activity, both on tomato (non-host) and cactus pear (host) plants in biological assays (Table 2 and Table 3), even at the lowest concentrations used (1.2 × 10^−4^ for **5,** 2.4 × 10^−4^ M for **2** and **6**). The metabolites **2**, **5**, and **6** thus proved to have almost the same spectrum of phytotoxic activity as they showed a comparable activity against host and non-host plants.

The results showed that (±)-botryoisocumarin A (**2**), (−)-terpestacin (**5**), and (+)-neoisocoumarin (**6**) were by far more phytotoxic than melleins (**1**, **3**, and **4**) when tested on cladodes of cactus pear and tomato leaves. Compounds **2** and **6** were the most active at concentrations in a range from 10^−3^ M to 10^−4^ M, inducing a necrosis area around the inoculation points in both host and non-host plants, followed by **5**. The ability of **2**, **5**, and **6** to induce large necrotic lesions on cactus pear cladodes suggests that these toxins may be involved in the scabby cankers disease syndrome. These results also indicate the ability of fungi in the Botryosphaeriaceae family to produce phytotoxins that are active against non-taxonomically related plants, such as cactus pear and tomato, and could explain the wide host spectrum of many species in the family. Moreover, it can be speculated that the allelopathic, antifungal, and antibacterial activity of terpestacin demonstrated in previous studies [25,44] might enhance the ecological fitness of *N. batangarum* and might explain the ability of this fungus to rapidly colonize the cactus pear cladode and sporulate on infected tissues [3] before they are invaded by other saprophytes or opportunistic weak pathogens of the plant biosphere. Due to its allelopathic activity, *N. batangarum* was indicated as a potential biocontrol agent [49,50]. In a recent review, Masi et al. [51] questioned the possibility of using terpestacin as a biopesticide because of its toxicity and stressed that, although the functions of this mycotoxin in nature have not been clearly established, its allelopathic activity would suggest a role in eliminating other microorganisms competing in the same environment.

## 3. Conclusions

(−)-(*R*)-Mellein, (−)-(3*R*,4*R*)- and (−)-(3*R*,4*S*)-4-hydroxymellein, (±)-botryoisocoumarin A, (−)-terpestacin, and (+)-neoisocoumarin were obtained for the first time as phytotoxins of *N. batangarum*. The absolute configuration of (+)-neoisocoumarin was determined by using the advanced Mosher’s method. Considering the phytotoxic activity on both host and non-host test plants, it can be deduced that all these metabolites were involved in the syndrome of scabby cankers disease of cactus pear, and like other Botryosphaeriaceae, *N. batangarum* has a wider host range than previously thought.

## 4. Materials and Methods

### 4.1. General Experimental Procedures

A Jasco P-1010 digital polarimeter (Jasco, Tokyo, Japan) was used to record the optical rotations in CHCl_3_, or as stated otherwise. Electrospray ionization (ESI) mass spectrometry and liquid chromatography/mass spectrometry (LC/MS) analyses were performed using an LC/MS TOF system Agilent 6230B (Agilent Technologies, Milan, Italy), HPLC 1260 Infinity. A Phenomenex (Bologna, Italy) Luna (C_18_ (2) 5 mm, 150 × 4.6 mm column) was used to perform the high-performance liquid chromatography (HPLC) separations. HPLC separation was carried out with the mobile phase used to elute the samples being MeCN-H_2_O 85:15 at a flow rate of 0.3 mL/min at 25 °C, injecting 10 mL of a solution of 1 ppm for the pure compounds. Bruker 400 Anova Advance (Karlsruhe, Germany) and Varian Inova 500 MHz (Palo Alto, CA, USA) instruments were used to record the ^1^H NMR spectra at 400 or 500 MHz in CDCl_3_, if not otherwise noted, at 298 °K. Column chromatography (CC) was performed using silica gel (Merck, Kieselgel 60, 0.063–0.200 mm). Preparative and analytical TLC were carried out on silica gel (Kieselgel 60, F_254_, 0.25 and 0.5 mm, respectively) plates (Merck, Darmstadt, Germany). The spots were visualized using the procedure previously described [47]. Sigma-Aldrich Co. (St. Louis, MO, USA) supplied all the reagents and the solvents.

### 4.2. Fungal Strain

The strains of *N. batangarum* used in this study were collected from infected host plant tissues on the islands of Favignana, Lampedusa, Linosa, and Ustica from 2013 to 2018 and maintained in potato dextrose agar (PDA, Fluka, Sigma-Aldrich Chemic GmbH, Buchs, Switzerland) and stored at 4 °C in the strain collection of the Dipartimento di Agricoltura, Alimentazione e Ambiente, Università di Catania, Catania, Italy. *N. batangarum* was obtained from scabby cankers on cladodes of cactus pear (*Opuntia ficus-indica* L.) recovered from minor islands of Sicily (Lampedusa, Linosa, Favignana, and Ustica). Four representative isolates, one from each island, were deposited at CBS-KNAW Biodiversity Centre, strains code nos. CBS143023, CBS143024, CBS143025, and CBS143026 [3].

### 4.3. Production, Extraction, and Isolation of Secondary Metabolites

The isolate CBS143023 (*Neofusicoccum batangarum*) was grown in liquid culture to obtain the liquid filtrate. In detail, the mycelium of fungal cultures, grown on potato dextrose agar for 5–7 days at 25 °C, was homogenized with sterile distilled water. Three milliliters of this suspension was distributed individually into 1 L Roux flasks containing 170 mL of modified Difco™ Czapeck-Dox (Benton, Dickinson and Company, Sparks, MD, USA) medium with 0.5% yeast and 0.5% malt extract (pH 5.75) and incubated at 25 °C for 28 days in the dark [15]. At harvest, the liquid cultures were filtered, initially with a double layer of gauze to reduce the fungal biomass, and subsequently with suction filters using porous membrane filter complexes Stericup Millipore^®^ (pore diameter = 0.22 μm). A total of 20 L of culture filtrate was obtained and stored at −20 °C until use. It was concentrated under reduced pressure at 35 °C to 1 L and extracted with EtOAc (3 × 1 L). The combined organic extracts were dried (Na_2_SO_4_) and evaporated under reduced pressure. The oily residue (1.84 g) was fractioned via column chromatography eluted with chloroform/*iso*-propanol (9:1), obtaining twelve groups of homogeneous fractions. The residues (24.4 mg and 23.1 mg) of the first and second fractions were combined and further purified using preparative TLC eluted with hexane/ethyl acetate (8.5:1.5), yielding two pure metabolites as amorphous solids that were identified as (−)-(*R*)-mellein (**1**, 8.6 mg, 0.7 mg/L, R*_f_* 0.4) and as (±)-botryoisocumarin A (**2**, 3.4 mg, 0.3 mg/L, R*_f_* 0.3). The residue (41.6 mg) of the fourth fraction of the first column was purified using preparative TLC eluted with petroleum ether:acetone 8:2, producing two pure metabolites identified as (−)-(3*R*,4*R*)-4-hydroxymellein (**3**, 2.4 mg, 0.2 mg/L, R*_f_* 0.3) and (−)-(3*R*,4*S*)-4-hydroxymellein (**4**, 3.6 mg, 0.3 mg/L, R*_f_* 0.4). The residue (123.4 mg) of the fifth fraction of the original column was further purified using column chromatography on silica gel eluted with dichloromethane/*iso*-propanol (9.5:0.5), obtaining eleven groups of homogeneous fractions. The residues (17.5 mg and 10.0 mg) of the seventh and eighth fractions were combined and purified using analytical TLC eluted with dichloromethane/*iso*-propanol (9.5:0.5), obtaining a pure metabolite as an amorphous solid, which was identified as (−)-terpestacin (**5**, 6.0 mg, 0.5 mg/L, R*_f_* 0.4). The residue (21.4 mg and 19.4 mg) of the fifth and sixth fractions were combined and purified using preparative TLC eluted with dichloromethane/*iso*-propanol (9.7:0.3), obtaining a pure compound identified as (+)-(3R*,4S*)-3,4-dihydro-4,5,8-trihydroxy-3methylisocoumarin, which was named (+)-neoisocumarin (**6**, 8.0 mg, 0.7 mg/L, R*_f_* 0.3).

*(−)-(*R*)-Mellein (**1**):* [α]^25^_D_ -90 (*c* 0.2 CH_3_OH); ^1^H NMR, δ: 11.03 (s, HO-8), 7.41 (t, *J* = 8.4 Hz, H-6), 6.89 (d, *J* = 8.4 Hz, H-7), 6.69 (d, *J* = 8.4 Hz, H-5), 4.74 (tq, *J* = 6.9 and 6.3 Hz, H-3), 2.93 (d, *J* = 6.9 Hz, H_2_-4), 1.53 (d, *J* = 6.3 Hz, Me-3). ESI MS (+) spectrum, *m*/*z* 179 [M + H] ^+^. These data are in agreement with those previously reported [13,17,18,19,20].

*(±)-Botryoisocoumarin (**2**):*^1^H NMR, δ: 11.03 (s, HO-8), 7.41 (t, *J* = 8.3 Hz, H-6), 6.89 (d, *J* = 8.3 Hz, H-7), 6.69 (d, *J* = 8.3, H-5), 3.40 (OMe), 3.23 (d, *J* = 16.0 Hz, H-4A) 3.16 (d, *J* = 16.0 Hz, H-4B), 1.69 (s, Me-C3). These data are in agreement with those previously reported [16]. ESI MS (+) spectrum, *m*/*z*: 439 [2M + Na] ^+^, 209 [M + H] ^+^, 176 [MH - MeOH] ^+^.

*(−)-(3*R*,4*R*)-4-Hydroxymellein (**3**):* [α]^25^_D_ -29.0 (*c* 1.2 CH_3_OH); ^1^H NMR, δ: 10.99 (s, HO-8), 7.55 (t, *J* = 7.0 Hz, H-6), 7.03 (d, *J* = 7.0 Hz, H-7), 7.00 (d, *J* = 7.0 Hz, H-5), 4.68 (br q, *J* = 7.0 Hz, H-3), 4.60 (br s, H-4), 1.53 (d, *J* = 7.0 Hz, Me-C3). ESI MS (+) spectrum, *m*/*z*: 195 [M + H] ^+^. These data are in agreement with the data previously reported [17,18,19,20,21].

*(−)-(3*R*,4*S*)-4-Hydroxymellein (**4**):* [α]^25^_D_ -27.0 (*c* 1.1 CH_3_OH); ^1^H NMR and ESI MS (+) data were very similar to those of **3**. These data are also in agreement with the data previously reported [17,18,19,20,21,22].

*(−)-Terpestacin (**5**):* [α]^25^_D_ -17.7 (*c* 0.4); ^1^H NMR, δ: 5.38 (m, H-12), 5.25 (dd, *J* = 10.6 and 5.4 Hz, H-2), 5.13 (m, H-6), 4.07 (dd, *J* = 9.7 and 3.6 Hz, H-10), 3.85 (dd, *J*=10.4 and 7.0 Hz, H-24A), 3.80 (dd, *J* = 10.4 and 5.5 Hz, H-24B), 2.66 (m, H-19), 2.36 (dd, *J* = 13.7 and 10.6 Hz, H-1A), 2.44 (d, *J* = 17.0 Hz, H-14), 2.26 and 2.11 (2H, both m, H_2_-5), 2.24 and 2.10 (2H, both m, H_2_-4), 2.18 and 1.78 (2H, both m, H_2_-8), 1.92 (m, H-13A), 1.75 (m, H-1B), 1.75 and 1.70 (both m, H_2_-9), 1.67 (s, Me-20), 1.64 (s, Me-21), 1.56 (s, Me-22), 1.29 (d, *J* = 7.3 Hz, Me-25), 0.99 (s, Me-23); ESI MS (+) spectrum, *m*/*z* 425 [M + Na] ^+^. These data are in agreement with those previously reported [23,24,25].

*(+)-Neoisocoumarin (**6**):* [α]^25^_D_ + 50 (*c* 0.4); ^1^H NMR, δ: 10.68 (s, HO-8), 7.16 (d, *J* = 9.0 Hz, H-6), 6.84 (d, *J* = 9.0 Hz, H-7), 5.03 (d, *J* = 4.1 Hz, H-4), 4.90 (dq, *J* = 6.7 and 4.4 Hz, H-3), 1.35 (d, *J* = 6.7 Hz, Me-C3); ESI MS (+) spectrum, *m*/*z* 443 [2M + Na]^+^, 233 [M + Na]^+^, 211 [M + H] ^+^, 193 [MH – H_2_O]^+^, 175 [MH - 2H_2_O]^+^. These data were very similar to that already reported [23].

*5,8-*O,O*-Dimethyl ether of (+)-neoisocoumarin (**7**):* To (+)-neoisocoumarin (**6**, 1 mg) in methanol (200 mL), diazomethane in ether solution was added in excess. The reaction performed overnight at 25 °C was stopped by evaporating the solvent under a N_2_ stream. The residue (1.2 mg) was purified using TLC, eluted with chloroform/*iso-*propanol 9.5:0.5 to yield **7** as a homogeneous oil (1.1 mg, R*_f_* 0.4). Derivate **7** had the following: ^1^H NMR, δ: 7.29 (d, *J* = 8.8 Hz, H-6), 7.07 (d, *J* = 8.8 Hz, H-7), 5.49 (d, *J* = 2.4 Hz, H-4), 4.48 (dq, *J* = 6.3 and 2.4 Hz, H-3), 3.90 and 3.88 (s, 3H each 2× OMe), 0.85 (d, *J* =6.3 Hz, Me-11); ESI MS (+) spectrum, *m*/*z* 499 [2M + Na] ^+^, 277 [M + K]^+^, 261 [M + Na]^+^, 239 [M + H]^+^, 221 [M + H-H_2_O]^+^.

*5-*O*-(*S*)-α-Methoxy-α-trifluoromethyl-α-phenylacetate (MTPA) ester of **7** (**8**).* To **7** (1.1 mg) in pyridine (100 mL), (*R*)-(−)-MPTA-Cl (10 mL) was added. The mixture was carried out for 1 h at 25°C and stopped by adding methanol and benzene. The mixture was evaporated using a N_2_ stream. The residue (1.3 mg) was purified using analytical TLC eluted with dichloromethane/*iso*-propanol (9.7:0.3), yielding **8** as a homogeneous oil (0.6 mg, R*_f_* 0.7). It had: ^1^H NMR, see Table 1; ESI MS (+) spectrum, *m*/*z* 477 [M + Na]^+^, 455 [M + H]^+^.

*5-*O*-(*R*)-α-methoxy-α-trifluoromethyl-*α*-phenylacetate (MTPA) ester of **6** (**9**):* To **7** (1.1 mg) in pyridine (100 mL), (*S*)-(+)-MPTA-Cl (10 mL) was added and the reaction was performed as previously reported. The crude residue (1.2 mg) was purified using analytical TLC eluted with dichloromethane/*iso*-propanol (9.7:0.3), producing **9** as a homogeneous oil (0.4 mg, R*_f_* 0.7). Compound **9** had: ^1^H NMR, see Table 1; ESI MS (+) spectrum, *m*/*z* 477 [M + Na]^+^, 455 [M + H]^+^.

### 4.4. Biological Assays

The metabolites isolated (**1**–**6**) were tested on young cladodes of host plant cactus pear (*Opuntia ficus-indica* (L.) Mill.) and on non-host tomato plant (*Solanum lycopersicum* L.) leaves. Pure metabolites were first dissolved in methanol, and then diluted with distilled water (final concentration of methanol, 4%) up to the desired concentrations. For each metabolite, 50 μL of the solution were pipetted into cladodes at concentrations of 0.05, 0.1, 0.25, 0.5, or 1 mg/mL. The phytotoxicity of the pure metabolites of *N. batangarum* was also tested using a puncture assay on tomato leaves. A droplet (10 μL) of each metabolite, at concentrations of 0.05, 0.1, 0.25, 0.5, or 1 mg/mL, was placed on the leaf lamina previously punctured by a needle. One cladode of a single plant of cactus pear and four leaves of tomato were used as replicates, and the test was repeated twice. Methanol (4% *v*/*v*) and sterile distilled water were used as controls. Symptoms of phytotoxicity of inoculated cladodes and leaves, kept in a climatic chamber under controlled conditions, were observed each day for 7 days. The size (mm^2^) of necrotic the area surrounding the punctures was measured.

## Figures and Tables

**Table 1:** ^1^H NMR data of 4-*O*-(*S*)- and 4-*O*-(*R*)-MTPA esters of 5,8-*O,O′*-dimetyl ether of (+)-neoisocoumarin (**8** and **9**, respectively).^1^

Position	8	9
	δ_H_ (*J* in Hz)	δ_H_ (*J* in Hz)
3	5.960 (1H) dq (2.2, 6.6)	5.958 (1H) dq (2.2, 6.6)
4	5.694 (1H) d (2.2)	5.829 (1H) d (2.2)
6	7.361 (1H) d (8.9)	7.368 (1H) d (8.9)
7	7.129 (1H) d (8.9)	7.141 (1H) d (8.9)
Me-C3O Me ^2^ OMe ^2^ OMe	1.092 (3H) d (6.6) 3.950(3H) s 3.888 (3H) s 3.574 (3H) s	0.982 (3H) d (6.6) 3.955 (3H) s 3.896 (3H) s 3.626 (3H) s
Ph	7.585–7.485 (5H) m	7.584–7.481 (5H) m

^1^ The chemical shifts are in δ values (ppm) from TMS (Tetramethylsilane). ^2^ These two signals could be exchanged.

**Table 2:** Phytotoxicity on tomato of **1**–**6** tested at various concentrations using the leaf puncture assay and evaluated based on the size of the necrotic lesions.

Compound	Concentration (M)	Mean Lesion Area (mm^2^) ^1^	Compound	Concentration (M)	Mean Lesion Area (mm^2^) ^1^
**1**	5.6 × 10^−3^	2.75 ± 0.68	**4**	5.2 × 10^−3^	3.14 ± 0.00
	2.8 × 10^−3^	2.36 ± 0.79		2.6 × 10^−3^	2.75 ± 0.68
	1.4 × 10^−3^	0.00 ± 0.00		1.3 × 10^−3^	0.00 ± 0.00
	5.6 × 10^−4^	0.00 ± 0.00		5.2 × 10^−4^	0.00 ± 0.00
	2.8 × 10^−4^	0.00 ± 0.00		2.6 × 10^−4^	0.00 ± 0.00
**2**	4.8 × 10^−3^	7.98 ± 1.28	**5**	2.5 × 10^−3^	5.10 ± 1.29
	2.4 × 10^−3^	7.46 ± 1.5		1.2 × 10^−3^	5.50 ± 1.04
	1.2 × 10^−3^	6.67 ± 1.5		6.2 × 10^−4^	4.81 ± 1.17
	4.8 × 10^−4^	6.80 ± 1.71		2.4 × 10^−4^	4.81 ± 1.3
	2.4 × 10^−4^	6.28 ± 1.03		1.2 × 10^−4^	4.61 ± 1.6
**3**	5.2 × 10^−3^	3.14 ± 0.00	**6**	4.8 × 10^−3^	7.98 ± 1.28
	2.6 × 10^−3^	2.36 ± 1.36		2.4 × 10^−3^	7.07 ± 1.26
	1.3 × 10^−3^	0.00 ± 0.00		1.2 × 10^−3^	6.67 ± 0.81
	5.2 × 10^−4^	0.00 ± 0.00		4.8 × 10^−4^	4.97 ± 1.55
	2.6 × 10^−4^	0.00 ± 0.00		2.4 × 10^−4^	5.36 ± 1.65

^1^ Mean of 12 replications (three plants and four leaves per plant).

**Table 3:** Phytotoxicity on cactus pear of **1**–**6** tested at various concentrations with the cladode puncture method and evaluated based on the size of the necrotic lesions.

Compound	Concentration (M)	Mean Lesion Area (mm^2^) ^1^	Compound	Concentration (M)	Mean Lesion Area (mm^2^) ^1^
**1**	5.6 × 10^−^^3^	3.14 ± 0.00	**4**	5.2 × 10^−^^3^	6.28 ± 1.56
	2.8 × 10^−^^3^	2.36 ± 0.79		2.6 × 10^−^^3^	2.36 ± 0.79
	1.4 × 10^−^^3^	2.75 ± 0.68		1.3 × 10^−^^3^	3.14 ± 0.00
	5.6 × 10^−^^4^	0.00 ± 0.00		5.2 × 10^−^^4^	0.00 ± 0.00
	2.8 × 10^−^^4^	0.00 ± 0.00		2.6 × 10^−^^4^	0.00 ± 0.00
**2**	4.8 × 10^−^^3^	11.00 ± 1.57	**5**	2.5 × 10^−^^3^	11.58 ± 1.57
	2.4 × 10^−^^3^	9.81 ± 1.75		1.2 × 10^−^^3^	7.07 ± 1.67
	1.2 × 10^−^^3^	11.78 ± 1.36		6.2 × 10^−^^4^	6.28 ± 1.32
	4.8 × 10^−^^4^	5.50 ± 0.79		2.4 × 10^−^^4^	3.53 ± 0.68
	2.4 × 10^−^^4^	7.07 ± 0.00		1.2 × 10^−^^4^	2.94 ± 1.40
**3**	5.2 × 10^−^^3^	3.14 ± 0.00	**6**	4.8 × 10^−^^3^	11.78 ± 1.60
	2.6 × 10^−^^3^	2.61 ± 0.93		2.4 × 10^−^^3^	9.62 ± 1.55
	1.3 × 10^−^^3^	3.14 ± 0.00		1.2 × 10^−^^3^	9.62 ± 1.55
	5.2 × 10^−^^4^	0.00 ± 0.00		4.8 × 10^−^^4^	4.51 ± 1.61
	2.6 × 10^−^^4^	0.00 ± 0.00		2.4 × 10^−^^4^	3.50 ± 0.68

^1^ Mean of eight replications (two cladodes from distinct plants and four punctures per each cladode).

## References

[1] Kieling R., Metzing D., Inglese P., Mondragon C., Nefzaoui A., Saenz C. (2017). Origin and Taxonomy of *Opuntia ficus-indica*. Crop Ecology, Cultivation and User of Cactus Pear.

[2] Ochoa M.J., Barbera G., Inglese P., Mondragon C., Nefzaoui A., Saenz C. (2017). History and economic and agro-ecological importance. Crop Ecology, Cultivation and Uses of Cactus Pear.

[3] Schena L., Surico G., Burruano S., Giambra S., Pane A., Evoli M., Cacciola S.O. (2018). First report of *Neofusicoccum batangarum* as causal agent of scabby cankers of Cactus pear (*Opuntia ficus-indica*) in minor islands of Sicily. Plant Dis..

[4] Phillips A.J.L., Alves A., Abdollahzadeh J., Slippers B., Wingfield M.J., Groenewald J.Z., Crous P.W. (2013). The Botryosphaeriaceae: Genera and species known from culture. Studies Mycol..

[5] Conforto C., Lima N.B., Garcete-Gómez J.M., Câmara M.P.S., Michereff S.J. (2016). First report of cladode brown spot in cactus prickly pear caused by *Neofusicoccum batangarum* in Brazil. Plant Dis..

[6] Netto M.S.B., Lima W.G., Correia K.G., Da Silva C.F.B., Thon M., Martins R.B., Miller R.N.G., Michereff F.J., Camâra M.P.F. (2017). Analysis of phylogeny, distribution and pathogenicity of Botryosphaeriaceae species associated with gummosis of *Anacardium* in Brasil, with a new species of *Lasiodipplodia*. Fungal Biol..

[7] Dissanayake A.J., Phillips A.J.L., Li X.H., Hyde K.D. (2016). Botryosphaeriaceae: Current status of genera and species. Mycosphere.

[8] Garcete-Gómez J.M., Conforto C., Domínguez-Monge S., Flores-Sánchez J.L.F., Mora-Aguilera G., Michereff S.J. (2017). Sample size for assessment of cladode brown spot in prickly pear cactus. Eur. J. Plant Pathol..

[9] Conforto C., Lima N.B., Silva F.J.A., Câmara M.P.S., Maharachchikumbura S., Michereff S.J. (2019). Characterization of fungal species associated with cladode brown spot on *Nopalea cochenillifera* in Brazil. Eur. J. Plant Pathol..

[10] Feijo F.M., Silva M.J.S., Nascimento A.D., Infante N.B., Ramos-Sobrinho R., Assunção I.P., Lima G.S.A. (2019). Botryosphaeriaceae species associated with the prickly pear cactus, *Nopalea cochinellifera*. Trop. Plant Pathol..

[11] Dissanayake A.J., Camporesi E., Hyde K.D., Phillips A.J.L., Fu C.Y., Yan J.Y., Li X.H. (2016). *Dothiorella* species associated with woody hosts in Italy. Mycosphere.

[12] Andolfi A., Maddau L., Cimmino A., Linaldeddu B.T., Basso S., Deidda A., Evidente A. (2014). Lasiojasmonates A–C, three jasmonic acid esters produced by *Lasiodiplodia* sp., a grapevine pathogen. Phytochemistry.

[13] Cimmino A., Cinelli T., Masi M., Reveglia P., da Silva M.A., Mugnai L., Evidente A. (2017). Phytotoxic lipophilic metabolites produced by grapevine strains of *Lasiodiplodia* species in Brazil. J. Agric. Food Chem..

[14] Reveglia P., Savocchia S., Billones-Baaijens R., Masi M., Cimmino A., Evidente A. (2019). Phytotoxic metabolites by nine species of Botryosphaeriaceae involved in grapevine dieback in Australia and identification of those produced by *Diplodia mutila*, *Diplodia seriata*, *Neofusicoccum australe* and *Neofusicoccum luteum*. Nat. Prod. Res..

[15] Masi M., Cimmino A., Reveglia P., Mugnai L., Surico G., Evidente A. (2018). Advances on fungal phytotoxins and their role in grapevine trunk diseases. J. Agric. Food Chem..

[16] Xu Y., Lu C., Zheng Z. (2012). A new 3,4-dihydroisocoumarin isolated from *Botryosphaeria* sp. F00741. Chem. Nat. Compd..

[17] Cole R.J., Cox R.H. (1981). Handbook of Toxic Fungal Metabolites;.

[18] Cabras A., Mannoni M.A., Serra S., Andolfi A., Fiore M., Evidente A. (2006). Occurrence, isolation and biological activity of phytotoxic metabolites produced *in vitro* by *Sphaeropsis sapinea*, pathogenic fungus of *Pinus radiata*. Eur. J. Plant Pathol..

[19] Evidente A., Punzo B., Andolfi A., Cimmino A., Melck D., Luque J. (2010). Lipophilic phytotoxins produced by *Neofusicoccum parvum*, a grapevine canker agent. Phytopathol. Mediterr..

[20] Abou-Mansour E., Débieux J.L., Ramírez-Suero M., Bénard-Gellon M., Magnin-Robert M., Spagnolo A., Serrano M. (2015). Phytotoxic metabolites from *Neofusicoccum parvum*, a pathogen of Botryosphaeria dieback of grapevine. Phytochemistry.

[21] Djoukeng J.D., Polli S., Larignon P., Abou-Mansour E. (2009). Identification of phytotoxins from *Botryosphaeria obtusa*, a pathogen of black dead arm disease of grapevine. Eur. J. Plant. Pathol..

[22] Devys M., Barbier M., Bousquet J.F., Kollmann A. (1992). Isolation of the new (−)-(3*R*, 4*S*)-4-hydroxymellein from the fungus *Septoria nodorum* Berk. Z. Naturforsch. C J. Biosci..

[23] Oka M., Iimura S., Tenmyo O., Sawada Y., Sugawara M., Ohkusa N., Oki T. (1993). Terpestacin, a new syncytium formation inhibitor from *Arthrinium sp*.. J. Antibiot..

[24] Trost B.M., Dong G., Vance J.A. (2007). A Diosphenol-based strategy for the total synthesis of (−)-terpestacin. J. Am. Chem. Soc..

[25] Cimmino A., Sarrocco S., Masi M., Diquattro S., Evidente M., Vannacci G., Evidente A. (2016). Fusaproliferin, terpestacin and their derivatives display variable allelopathic activity against some Ascomycetous fungi. Chem. Biodivers..

[26] Yang J.X., Chen Y., Huang C., She Z., Lin Y. (2011). A new isochroman derivative from the marine fungus *Phomopsis sp*. (No. ZH-111). Chem. Nat. Compd..

[27] Krohn K., Bahramsari R., Flörke U., Ludewig K., Kliche-Spory C., Michel A., Antus S. (1997). Dihydroisocoumarins from fungi: Isolation, structure elucidation, circular dichroism and biological activity. Phytochemistry.

[28] Rukachaisirikul V., Arunpanichlert J., Sukpondma Y., Phongpaichit S., Sakayaroj J. (2009). Metabolites from the endophytic fungi *Botryosphaeria rhodina* PSU-M35 and PSU-M114. Tetrahedron.

[29] Salazar A., Rios I. (2010). Sustainable Agriculture: Technology, Planning and Management.

[30] Ramírez-Suero M., Bénard-Gellon M., Chong J., Laloue H., Stempien E., Abou-Mansour E., Bertsch C. (2014). Extracellular compounds produced by fungi associated with Botryosphaeria dieback induce differential defence gene expression patterns and necrosis in *Vitis vinifera* cv. Chardonnay cells. Protoplasma.

[31] Cimmino A., Maddau L., Masi M., Linaldeddu B.T., Evidente A. (2019). Secondary metabolites produced by *Sardiniella urbana*, a new emerging pathogen on European hackberry. Nat. Prod. Res..

[32] Bestmann H.J., Kern F., Schäfer D., Witschel M.C. (1992). 3, 4-dihydroisocoumarins, a new class of ant trail pheromones. Angew. Chem..

[33] Kern F., Klein R.W., Janssen E., Bestmann H.J., Attygalle A.B., Schäfer D., Maschwitz U. (1997). Mellein, a trail pheromone component of the ant *Lasius fuliginosus*. J. Chem. Ecol..

[34] Manigaunha A., Ganesh N., Kharya M.D. (2010). Morning glory: A new thirst in-search of *de-novo* therapeutic approach. Int. J. Phytomed..

[35] Herzner G., Schlecht A., Dollhofer V., Parzefall C., Harrar K., Kreuzer A., Ruther J. (2013). Larvae of the parasitoid wasp *Ampulex compressa* sanitize their host, the American cockroach, with a blend of antimicrobials. Proc. Natl. Acad. Sci. USA.

[36] Cimmino A., Maddau L., Masi M., Linaldeddu B.T., Pescitelli G., Evidente A. (2017). Fraxitoxin, a new isochromanone isolated from *Diplodia fraxini*. Chem. Biodivers..

[37] Masi M., Maddau L., Linaldeddu B.T., Scanu B., Evidente A., Cimmino A. (2018). Bioactive metabolites from pathogenic and endophytic fungi of forest trees. Curr. Med. Chem..

[38] Höller U., König G.M., Wright A.D. (1999). Three new metabolites from marine-derived fungi of the genera *Coniothyrium* and *Microsphaeropsis*. J. Nat. Prod..

[39] Dai J.R., Carté B.K., Sidebottom P.J., Sek Yew A.L., Ng S.B., Huang Y., Butler M.S. (2001). Circumdatin G, a new alkaloid from the fungus *Aspergillus ochraceus*. J. Nat. Prod..

[40] Zhao J.H., Zhang Y.L., Wang L.W., Wang J.Y., Zhang C.L. (2012). Bioactive secondary metabolites from *Nigrospora sp*. LLGLM003, an endophytic fungus of the medicinal plant *Moringa oleifera* Lam. World J. Microbiol. Biotechnol..

[41] Cimmino A., Masi M., Evidente M., Superchi S., Evidente A. (2015). Fungal phytotoxins with potential herbicidal activity: Chemical and biological characterization. Nat. Prod. Rep..

[42] Evidente M., Cimmino A., Zonno M.C., Masi M., Berestetskyi A., Santoro E., Evidente A. (2015). Phytotoxins produced by *Phoma chenopodiicola*, a fungal pathogen of *Chenopodium album*. Phytochemistry.

[43] Ju Z., Lin X., Lu X., Tu Z., Wang J., Kaliyaperumal K., Liu Y. (2015). Botryoisocoumarin A, a new COX-2 inhibitor from the mangrove *Kandelia candel* endophytic fungus *Botryosphaeria sp*. KcF6. J. Antibiot..

[44] Liu Y., Li X.M., Meng L.H., Wang B.G. (2015). Polyketides from the marine mangrove-derived fungus *Aspergillus ochraceus* MA-15 and their activity against aquatic pathogenic bacteria. Phytochem. Lett..

[45] Fredimoses M., Zhou X., Ai W., Tian X., Yang B., Lin X., Liu Y. (2015). Westerdijkin A, a new hydroxyphenylacetic acid derivative from deep sea fungus *Aspergillus westerdijkiae* SCSIO 05233. Nat. Prod. Res..

[46] Liu D., Li X.M., Li C.S., Wang B.G. (2013). Sesterterpenes and 2*H*-pyran-2-ones (= α-pyrones) from the mangrove-derived endophytic fungus *Fusarium proliferatum* MA84. Helv. Chim. Acta.

[47] Masi M., Meyer S., Górecki M., Pescitelli G., Clement S., Cimmino A., Evidente A. (2018). Phytotoxic activity of metabolites isolated from *Rutstroemia sp*. n., the causal agent of bleach blonde syndrome on cheatgrass (*Bromus tectorum*). Molecules.

[48] Cimmino A., Masi M., Evidente M., Superchi S., Evidente A. (2017). Application of Mosher’s method for absolute configuration assignment to bioactive plants and fungi metabolites. J. Pharm. Biomed..

[49] Shetty K.G., Minnis A.M., Rossman A.Y., Jayachandran K. (2011). The Brazilian peppertree seedborne pathogen, *Neofusicoccum batangarum*. Biol. Control.

[50] Dawkins K., Esiobu N. (2016). Emerging insights on Brazilian pepper tree (*Schinus terebinthifolius*) invasion: The potential role of soil microorganisms. Front. Plant Sci..

[51] Masi M., Nocera P., Reveglia P., Cimmino A., Evidente A. (2018). Fungal metabolites antagonists towards plant pests and human pathogens: Structure-activity relationship studies. Molecules.

